# Identification of Novel *Streptomyces* sp. BPTC-684 as a Biocontrol Agent Against Challenging Maize Root Rot Caused by *Fusarium verticillioides*

**DOI:** 10.3390/microorganisms14040818

**Published:** 2026-04-02

**Authors:** Tran Van Chi, Nguyen Trinh Hoang Anh, Tuan Manh Nguyen

**Affiliations:** 1 Institute of Biotechnology—Food Technology, Thai Nguyen University of Agriculture and Forestry, 251-210 Quyet Thang, Thai Nguyen 25000, Vietnam; 2 Ministry of Science and Technology, 18 Nguyen Du, Ha Noi 100000, Vietnam; 3 Institute of Life Sciences, Thai Nguyen University, 251-210 Quyet Thang, Thai Nguyen 25000, Vietnam; 4 Faculty of Agricultural Technology, Thai Nguyen University of Agriculture and Forestry, 251-210 Quyet Thang, Thai Nguyen 25000, Vietnam

**Keywords:** biocontrol agent, *Fusarium verticillioides*, maize root rot, secondary metabolites, *Streptomyces* species, sustainable agriculture

## Abstract

Maize (*Zea mays* L.) cultivation is severely affected by *Fusarium verticillioides*, a highly adaptable systemic pathogen that causes serious yield losses, reduces grain quality, and produces toxic fumonisin, posing significant health risks to humans and livestock. A biological control approach to combating it was investigated. *Streptomyces* sp. BPTC-684 showed strong inhibitory activity (53.11%) against *F. verticillioides* BNGO-16, isolated from a diseased tissue sample. Based on physiological and biochemical characteristics, 16S rRNA gene sequencing, average nucleotide identity, and digital DNA–DNA hybridization, strain BPTC-684 is considered a candidate new species belonging to the genus *Streptomyces*. In silico analysis of *Streptomyces* sp. BPTC-684 showed that it expresses diverse biosynthetic gene clusters encoding potential bioactive compounds, notably antibiotics (kinamycin, antimycin, fuelimycins A-C, hangtaimycin, and deoxyhangtaimycin) and siderophores (desferrioxamines B and E). In addition, plant growth-promoting behaviors, such as indole-3-acetic acid production; phosphate solubilization; and the production of extracellular lytic enzymes that degrade cellulose, chitin, proteins, amylose, and xylan, were also discovered in *Streptomyces* sp. BPTC-684. The pot experiments demonstrated that plant height, fresh weight, and dry root weight were increased in strain BPTC-684 by 37.88%, 132.50%, and 223.81%, respectively, compared to *F. verticillioides* BNGO-16 on the 15th day of infection. These findings suggest that *Streptomyces* sp. BPTC-684 is a promising biological control agent for inhibiting fungal diseases and promoting maize growth.

## 1. Introduction

Maize (*Zea mays* L.) is the most widely grown crop worldwide, playing a crucial role in ensuring food security and serving as an essential raw material for various industries. According to the Foreign Agricultural Service [1], global maize production is projected to reach 1.23 billion metric tons, accounting for approximately 44% of total major grain production in 2024. In Vietnam, with its tropical climate favorable for agricultural development, maize contributes about 12% to the country’s gross domestic product (GDP) [2]; it is regarded as the second-most-important food crop and provides the greatest economic value in Vietnam. Both the total area and production of maize have increased annually, from 0.43 million hectares and 0.67 million tons in 1990 to 0.77 million hectares and 4.42 million tons in 2022 [3,4]. Maize is an indispensable food source and a key ingredient in the production of animal feed for the growing livestock and poultry industry. The role of maize is even more evident in the midland and mountainous regions of Northern Vietnam, where the terrain is mainly hilly; cultivation must take place on sloping land, and water resources are difficult to control, making rice cultivation difficult [3,4].

However, tropical climates pose significant challenges in controlling plant diseases and pests caused by fungi, viruses, and bacteria. Among these, *F. verticillioides* is the main causative agent of infectious wilt and rot disease in maize [5,6], resulting in a potential 30–50% reduction in yield [7]. Furthermore, *F. verticillioides* produces dangerous mycotoxins (toxic metabolites with low molecular weight), notably fumonisin B1, which easily contaminates the food chain, posing serious health risks to humans and animals, such as cancer, liver damage, and immunosuppression [8,9]. Furthermore, fumonisin B1 is stable at normal boiling temperatures, only being destroyed at very high temperatures [10,11], which further increases the risk of toxin contamination in the food chain from raw materials contaminated with *F. verticillioides* when they are used in food and animal feed processing.

*F. verticillioides* is widely known as a soil-borne fungus that infects maize through the root system or seeds planted in contaminated soil. Not only is it capable of thriving in a variety of conditions, such as drought and high temperatures [6,12,13], but it also produces spores, including microconidia and macroconidia, allowing for widespread dispersal and long-term survival under adverse conditions. This hampers the effectiveness of crop rotation and soil improvement measures in agriculture. While chemical pesticides can effectively control *F. verticillioides*, their long-term use poses risks of pesticide residue in products and the environment, increased resistance to pesticides, and the elimination of non-target beneficial insects [14,15,16,17].

Recent studies have shown that using microorganisms that produce characteristic metabolites, such as antifungals, to control plant fungal diseases not only supports improved nutrient absorption and enhances disease resistance pathways but also provides a safe, environmentally friendly solution [18,19]. Microorganisms inhibit plant fungal diseases by producing antifungal substances, extracellular lytic enzymes, and siderophores [6,20]. To date, a diverse range of species for controlling plant fungal diseases have been reported, with the genus *Streptomyces* as a potential source of biomaterials [20,21]. The aim of this study was to identify the fungus causing maize root rot and to gain insights into the antagonistic activity of *Streptomyces* sp. BPTC-684 through in vitro and genome analysis.

## 2. Materials and Methods

### 2.1. Sampling, Isolation, and Identification of Pathogenic Fungi

**Figure 1:**
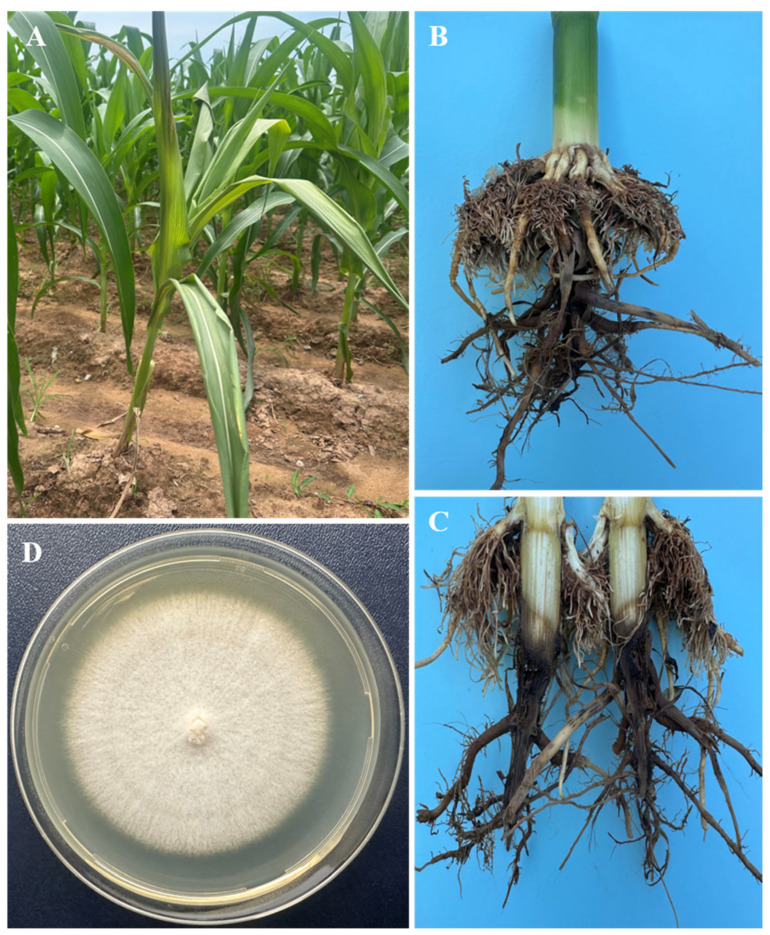
Morphology of corn infected with fungal disease. Diseased corn in the field (**A**); External (**B**) and internal (**C**) symptoms of corn root rot; Colony morphology on the front sides (**D**) of strain BNGO-16 on PDA medium at 25 °C, 5 days of incubation.

A diseased maize root sample (Figure 1A–C) was collected in Bac Kan, Vietnam (GPS 22°32′17″ N 105°59′47″ E), in October 2024. The pathological tissue sample was washed three times in sterile distilled water, cut, and surface-disinfected as described by Wang et al. [5]. Then, the pieces were placed on potato dextrose agar (PDA) medium (200 g of potato, 10 g of anhydrous glucose, 15 g of agar, 1 L of distilled water, pH 5.6) supplemented with oxytetracycline at a final concentration of 50 mg/L. The plates were cultured at 25 °C for 5 days in the dark. Subsequently, several subcultures were performed on a clean PDA plate to obtain pure mycelia, which were then stored at a final glucose concentration of 10% at −70 °C. Pure mycelium was cultured in potato dextrose broth (PDB: 200 g of potato, 10 g of glucose, 1 L of distilled water, pH 5.6) for 24 h at 130 rpm for DNA extraction. The Quick-DNA Fungal/Bacterial Midiprep Kit (Zymo Research, Irvine, CA, USA) was used to extract genomic DNA following the manufacturer’s instructions.

The Internal Transcribed Spacer (*ITS*) and Translation Elongation Factor 1-alpha (*TEF-1α*) genes were used to identify the isolated fungus via PCR. The primer pair ITS1F TCCGTAGGTGAACCTGCGG and ITS4R TCCTCCGCTTATTGATATGC was used for the ITS gene [22], and the primer pair EF1-728F CATCGAGAAGTTCGAGAAGG-3′ and EF2-986R TACTTGAAGGAACCCTTACC was used for the *TEF-1α* gene [23]. The PCRs were prepared in final volumes of 25 µL containing 10 ng of genomic DNA, with 35 thermal cycles as described by Wang et al. [5], using annealing temperatures of 58 °C for *ITS* and 53 °C for *TEF-1α*. The PCR products were sequenced at Macrogen Inc. (Synapse, Singapore). The *ITS* and *TEF-1α* gene sequences were compared with *Fusarium* species published in the FUSARIOID-ID database (https://www.fusarium.org; accessed on 12 February 2026). The *ITS* and *TEF-1α* gene sequences of related species were obtained from the FUSARIOID-ID database, and multi-sequence alignment was performed using the CLUSTAL X2 [24]. The neighbor-joining method [25] was used in the MEGA v.12 program [26] to reconstruct the phylogenetic tree; bootstrap values were based on 1000 repeats [27].

### 2.2. Isolation of Actinomycetes

Ten rhizosphere soil samples from maize grown in hilly areas in Cao Bang, Vietnam, were collected in October 2022. Soil samples (10 g), after drying at room temperature and removing plant biomass and stones, were diluted in 90 mL of 0.85% (*w*/*v*) NaCl, and further diluted to 10^−6^. The suspension (100 µL) was spread onto International *Streptomyces* Project Medium 4 (ISP 4: 10 g of difco soluble starch, 2 g of CaCO_3_, 1 g of K_2_HPO_4_, 1 g of MgSO_4·_7H_2_O, 1 g of NaCl, 2 g of (NH_4_)_2_SO_4_, 1 mg of FeSO_4_·7H_2_O, 1 mg of MnCl_2_·4H_2_O, 1 mg of ZnSO_4_·7H_2_O, 15 g of agar, 800 mL of distilled water) [28] with a slight modification, adding 200 mL of soil extract [29] to obtain a greater diversity of growing Actinomycetes species. Briefly, the soil extract was prepared as follows: 1000 g of dry maize rhizosphere soil was extracted in 80% methanol, then the methanol was removed using a vacuum rotary evaporator at 40 °C. The residue was dissolved in 200 mL of sterile distilled water and filtered through a 0.22 µm nitrocellulose filter (GSWP04700; Merck Millipore Ltd., Darmstadt, Germany). This filtrate (200 mL) was added to the sterilized ISP 4 medium. A 50 mg/mL of cycloheximide stock solution filtered through a 0.2 µm membrane was also supplemented with ISP4 medium at a final concentration of 50 mg/L after sterilization at approximately 50 °C to inhibit fungal contamination from the collected soil samples. The final pH of the modified medium was 7.0 ± 0.2. The plates were cultured at 28 °C for 2 weeks under aerobic conditions. Colonies were purified by subculture and stored at −70 °C in 20% (*v*/*v*) glycerol.

### 2.3. Screening of Antifungal Isolates

*F. verticillioides* BNGO-16 was activated on PDB medium at 28 °C for 10 days, with shaking at 150 rpm. Spores of *F. verticillioides* BNGO-16 were collected by centrifugation at 3000 rpm and 4 °C, for 15 min. The residue was washed in sterile distilled water and filtered through sterile gauze to remove mycelium and hypha debris. The spore suspension was adjusted to a final concentration of 1 × 10^6^ CFU/mL [30]. The isolated Actinomycetes were refreshed in R2A medium (0.5 g of yeast extract, 0.5 g of proteose peptone, 0.5 g of casamino acids, 0.5 g of glucose, 0.5 g of starch, 0.3 g of Na-pyruvate, 0.3 g of K_2_HPO_4_, 0.05 g of MgSO_4_·7H_2_O, 15 g of agar, 1 L of distilled water, pH 7.2) for 3 days at 28 °C. Antifungal screening was performed by spot inoculation on PDA medium. The isolates were spot-inoculated onto a PDA control medium containing 100 µL of *F. verticillioides* BNGO-16 spore suspension at a concentration of 1 × 10^6^ CFU/mL that had been spread. The plates were cultured at 25 °C for 7 days. The antifungal activity of the isolates was determined using the formula D–d, where D is the average diameter of the antifungal zone (mm) and d is the diameter of the isolated actinomycete (mm). Simultaneously, the percentage inhibition of fungal growth was determined using the agar plug method (5 mm in diameter) and then calculated according to the description of Zygadlo et al. [31] after 7 days of incubation at 25°C via the formula PI (%) = [(D0 − D1)/D0] × 100, where D0 is the average diameter of the untreated fungus, and D1 is the diameter of the fungus treated with the antifungal isolate. The experiments were carried out with three biological replications. Based on the antifungal zone results, strain BPTC-684 was selected for further studies.

### 2.4. Morphological and Biochemical Characteristics of Strain BPTC-684

The phenotypic characteristics of strain BPTC-684 were determined according to the description by Shirling & Gottlieb [32] using Czapek–Dox agar, Potato dextrose agar, Nutrient agar, Trypticase soy agar (TSA), Yeast extract–malt extract agar (ISP 2), Oatmeal agar (ISP 3), Inorganic salts–starch agar (ISP 4), Glycerol–asparagine agar (ISP 5), and Peptone Yeast Extract Iron Agar (ISP 6) at 28 °C for 21 days. The morphology of the spores of the isolate cultured on ISP 4 and incubated at 28 °C for 21 days was observed using a scanning electron microscope (EVO MA 10, ZEISS, Jena, Germany). Simultaneously, strain BPTC-684 was grown at various temperatures (10, 15, 20, 25, 28, 30, 37, 40, and 45 °C), pHs (4.0–10.0; at 0.5 unit intervals), and NaCl concentrations (1–10%; at 0.5% unit intervals) on Trypticase soy agar medium.

The siderophore produced by strain BPTC-684 was detected on Chrome Azurol S medium (MP, Biomedicals, Irvine, CA, USA) at 28 °C over 14 days of incubation. The siderophore production index (SPI) was determined from the orange-yellow halo formed around the colony as described by Afridi et al. [33] as follows: SPI = (halo color zone + colony)/colony diameters.

The indole-3-acetic acid (IAA) product was determined according to the description by Glickmann and Dessaux [34]. Briefly, strain BPTC-684 was cultured in 150 mL Erlenmeyer flasks containing 30 mL of TSB medium supplemented with 0.1% L-tryptophan (*w*/*v*), with shaking at 150 rpm, at 28 °C for 7 days. The cell biomass was removed by centrifugation, and the culture was mixed with Salkowski reagent at a 2:1 (*v*/*v*) ratio in a capped glass tube. The suspension was covered with foil, mixed well, and kept at room temperature for 30 min. A color change from yellow to pink was considered positive. The IAA content (µg/mL) in the suspension was determined at a wavelength of 530 nm using a UV-VIS spectrophotometer (BioMate 3, Thermo Fisher Scientific, Waltham, MA, USA), and an IAA calibration curve across concentrations of 0–100 µg/mL was constructed.

The extracellular lytic activities of strain BPTC-684 were also assayed, including cellulose degradation using carboxymethyl cellulose medium (CMC: 1 g of (NH_4_)_2_SO_4_, 1 g of MgSO_4_·7H_2_O, 1 g of CaCl_2_·2H_2_O, 0.2 g of FeCl_3_, 1 g of K_2_HPO_4_, 2 g of casitone, 15 g of carboxymethyl cellulose (Sigma-Aldrich, St. Louis, MO, USA), 6 g of agar, 1 L of distilled water). For chitin degradation, the medium used contained 10 g of chitin ((C_8_H_13_NO_5_)_n_; Sigma-Aldrich), 6 g of Na_2_HPO_4_, 3 g of KH_2_PO4, 1 g of NH_4_Cl, 20 g of NaCl, 0.05 g of yeast extract, 0.5 g of MgSO_4_·7H_2_O, 15 g of agar, and 1 L of distilled water. These culture plates were incubated at 28 °C for 7 days, and the halo color was recorded as a clear zone or as a 1% Congo red stain. Each experiment was carried out with three biological replications.

### 2.5. Classification of Strain BPTC-684 Based on 16S rRNA Gene and Whole Genome Sequencing

#### 2.5.1. Identification of 16 rRNA Gene Sequencing

Strain BPTC-684 was cultured in Tryptic soy broth at 28 °C for 2 days, with shaking at 150 rpm. DNA was extracted using the Quick-DNA Fungal/Bacterial Midiprep Kit (Zymo Research, Irvine, CA, USA). The two primer pairs used were 27F-AGAGTTTGATCMTGGCTCAG and 1492R TACGGYTACCTTGTTACGACTT [35]. A final PCR volume of 50 µL was prepared containing 80 ng of genomic DNA. The PCR amplification was performed for 25 cycles, with an annealing temperature of 55 °C [36]. The PCR products were sequenced at Macrogen Inc. (Synapse, Singapore). The 16S rRNA gene sequence of strain BPTC-684 was compared with the EzBioCloud Database (https://www.ezbiocloud.net/; accessed on 16 February 2026). Simultaneously, a phylogenetic tree based on the 16S rRNA gene sequence of BPTC-684 and its closest species was constructed in MEGA v.12 [26]. The 16 rRNA gene sequencing was performed with three replications. The accession number for the 16S rRNA gene sequence of strain BPTC-684 is OQ921823.

#### 2.5.2. Characteristic and Taxonomic Whole Genome Sequencing

The genome of strain BPTC-684 was assembled using a combination of Illumina MiniSeq (Illumina, San Diego, CA, USA) and Oxford Nanopore Technologies (ONT, Oxford, UK). Adapters and low-quality nucleotides were removed from the Illumina and ONT sequences using Fastp v0.23.1 [37] and Filtlong v0.2.1 [38]. Flye v2.9 [39] was used for de novo assembly of the genomic DNA sequence. Next, newly assembled contigs were corrected using Medaka v1.4.3 and POLCA (MaSuRCA v4.0.9). Circlator v1.5.5 [40] was used for splicing and identifying cyclic contigs. The whole genome sequence was subjected to rapid annotation using a subsystem technology tool kit (RASTtk) [41]. The genome sequencing was completed with three replications. The whole genome sequencing accession number for strain BPTC-684 is CP124863. Then, secondary metabolite gene clusters were identified using antiSMASH v.8.0.4 software (https://antismash.secondarymetabolites.org/; accessed on 10 February 2026) [42]. Simultaneously, the taxonomy of strain BPTC-684 and its closest type strains based on genomes was simultaneously confirmed via digital DNA–DNA hybridization (dDDH) using the Genome-to-Genome Distance Calculator v3.0 (https://ggdc.dsmz.de/ggdc.php; accessed on 7 February 2026) [43], and Average Nucleotide Identity (ANI) values were determined using OrthoANI v8.0, accessed on 10 February 2026 [44] and ANI based on BLAST v2.2.29^+^ (ANIb), accessed on 10 February 2026 [45].

### 2.6. Plant Growth Promotion in Pot Experiment

Native glutinous corn seeds from Bac Kan, Vietnam, were used in this study. These corn seeds were treated in a 0.75% (*w*/*v*) NaOCl solution for 30 min. The NaOCl was then removed using sterile distilled water [46]. Two-day-old germinated corn was prepared and tested to evaluate the plant growth-promoting ability of *Streptomyces* sp. BPTC-684 as described by Figueroa-López et al. [47], with the following changes: spores at a density of 1 × 10^6^ CFU/mL of the *F. verticillioides* BNGO-16 and BPTC-684 were prepared separately. First, each germinated corn seed was planted individually in a separate pot containing 1 kg of a 4:1 (*w*/*w*) mixture of sandy loam and well-rotted manure. Next, 50 mL of suspension containing 1 × 10^6^ CFU/mL of either *F. verticillioides* BNGO-16 or *Streptomyces* sp. BPTC-684 was added to the experimental pots. The experiments included a control (without added *F. verticillioides* BNGO-16 or *Streptomyces* sp. BPTC-684 as control), *F. verticillioides* BNGO-16, and *Streptomyces* sp. BPTC-684 + *F. verticillioides* BNGO-16. These pots were placed in a greenhouse with temperatures maintained at 25–30 °C and a natural light cycle. Distilled water was applied regularly once every 3 days. The maize growth was determined on the 15th day in terms of plant height (cm), fresh plant weight (g), and dried root weight (g). The experiments were established with a completely randomized design with three replications, each using ten plants in a pot experiment.

### 2.7. Statistical Analysis

The results of this study are presented as means ± standard deviations, analyzed using JASP v0.95.4 software. A significance level of *p* < 0.05 was used to determine statistical significance.

## 3. Results

### 3.1. Identifying Pathogenic Fungi in Infected Corn Growing in the Field

The corn, collected from the field, showed signs of wilting, yellowing leaves, and rotting (Figure 1A), and crumbling roots (Figure 1B,C). Five fungal strains were detected from the tissue samples, but they showed no differences in colony morphology characteristics or growth rate on PDA medium. These isolates were designated as strain BNGO-16. Colonies of strain BNGO-16 were white in color, with a cottony to porous surface, smooth margin, and growth rate of 4.75 ± 0.68 mm/day (Figure 1D).

**Figure 2:**
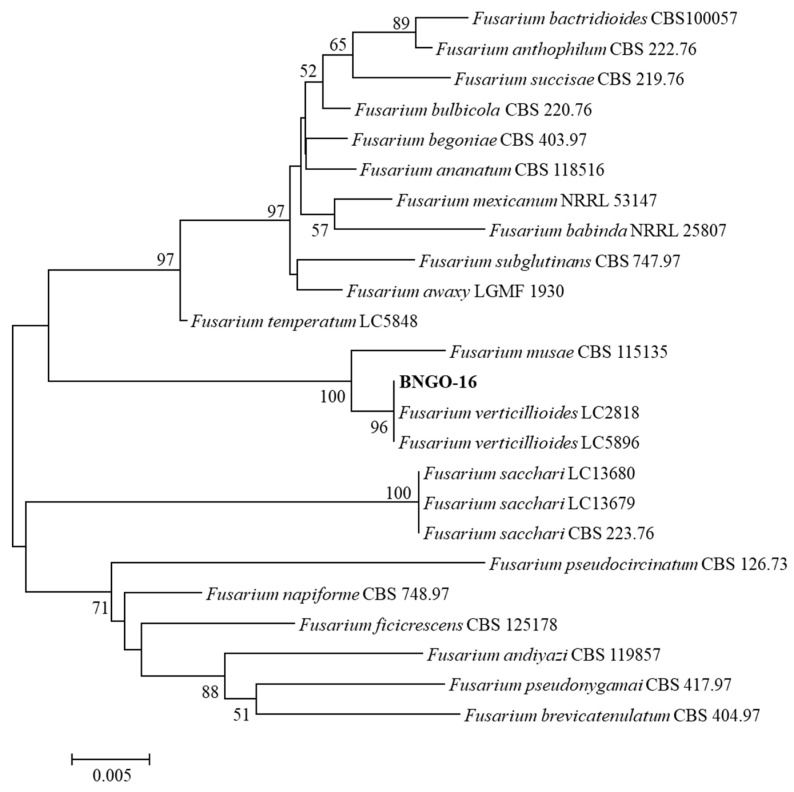
Neighbour-joining tree based on a combination of *ITS* and *TEF-1α* of strain BNGO-16 and closely related members of the genus *Fusarium*. Note: Numbers at the nodes represent level of bootstrap support (%) based on a neighbour-joining analysis of 1000 resampled datasets. Only bootstrap values greater than 50% are mentioned. Bar, 0.005 substitutions per site.

To determine the taxonomy of strain BNGO-16, two genes—*ITS* (accession number PX475066; length of 559 bp) and *TEF-1α* (PX724859; 673 bp)—were used. The results of *ITS* and *TEF-1α* sequencing were compared using the FUSARIOID-ID database v3.0, revealing that strain BNGO-16 belongs to the genus *Fusarium*. Simultaneously, a neighbor-joining tree was constructed by associating the *ITS* and *TEF-1α* within members of the genus *Fusarium*. The results indicated that strain BNGO-16 was monohybridized from *F. verticillioides* LC2818 and *F. verticillioides* LC5896, with a bootstrap value of 96%. The taxonomic position of strain BNGO-16 was distinct from that of other *Fusarium* species (Figure 2). Therefore, strain BNGO-16 is identified as *F. verticillioides*.

### 3.2. Screening of Antifungal Isolates and Morphological Strain BPTC-684

**Figure 3:**
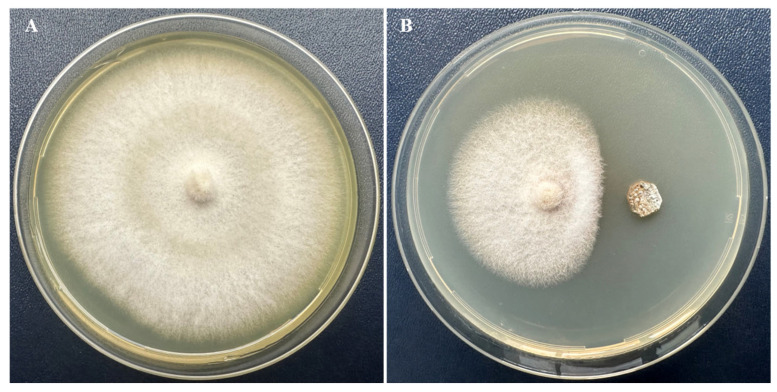
Activity against *F. verticillioides* BNGO-16 of strain BPTC-684 at 25 °C, 7 days of incubation. (**A**): control and (**B**): strain BPTC-684. Note: Strain BPTC-684 produced bioactive substances that diffused into the medium, inhibiting 53.11 ± 2.41% of the fungus BNGO-16. Data is determined as mean ± standard deviation (SD).

From 10 corn rhizosphere soil samples, 19 Actinomycete species exhibiting growth inhibition against *F. verticillioides* BNGO-16 were isolated using the spot culture method. Among these, strain BPTC-684 showed the strongest antifungal activity with an inhibition zone of 53.11 ± 2.41% (Figure 3).

**Figure 4:**
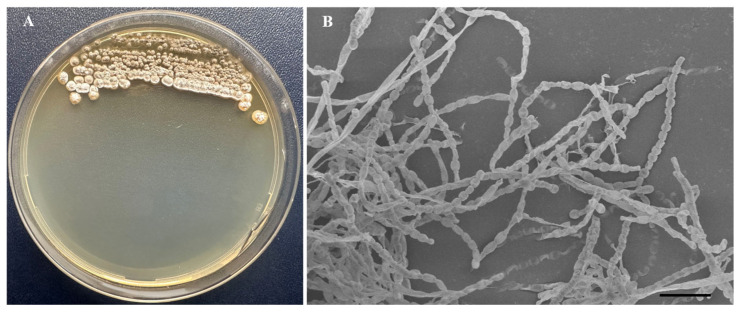
Morphology characteristics of strain BPTC-684 grown on inorganic salts starch agar (ISP 4) at 28°C for 21 days. (**A**): Colonies and (**B**): Scanning electron micrograph of spore chain morphology of strain BPTC-684 at 3000× magnification with a 5 µm scale bar.

Strain BPTC-684 was capable of growing in a variety of media, including Czapek–Dox agar, potato dextrose agar, nutrient agar, trypticase soy agar, ISP 2, ISP 3, ISP 4, ISP 5, and ISP 6 (optimum nutrient agar, ISP 2, ISP 3, ISP 4, ISP 5, and ISP 6). Spores formed on potato dextrose agar, ISP 2, ISP 3, and ISP 4, with light gray coloration on potato dextrose agar, ISP 3, and ISP 4 (Figure 4A). Reverse mycelium and diffusible pigment coloration appeared on trypticase soy agar, ISP 2, ISP 3, ISP 4, ISP 5, and ISP 6 after 21 days of cultivation at 28 °C (Table S1). On ISP 4, strain BPTC-684 produced straight-to-rectiflexible spore chains and elliptical or short, rod-shaped spores with smooth surfaces (Figure 4B). The isolate grew at 20–40 °C and pH 4–9, and was resistant to 7% NaCl. Notably, it produced IAA and siderophore with activities of 61.74 ± 2.48 µg/mL and 5.05 ± 0.11, respectively. Extracellular hydrolytic enzymes, including cellulase, protease, and chitinase, were also produced by the isolate. Strain BPTC-684 was able to digest L-arabinose, D-xylose, D-fructose, cellulose, and L-tyrosine as carbon and nitrogen sources, respectively (Table 1).

### 3.3. Cultural and Molecular Taxonomy of Strain BPTC-684

Cultural characteristics of strain BPTC-684 and closely related *Streptomyes* species were compared, revealing differences in aerial mycelium color, substrate mycelium color, and production of soluble pigment on specialized ISP 2–6 media. Notably, soluble pigment was detected in strain BPTC-684 on ISP 2, ISP 4, and ISP 5 media, while other species did not produce (Table 2).

**Figure 5:**
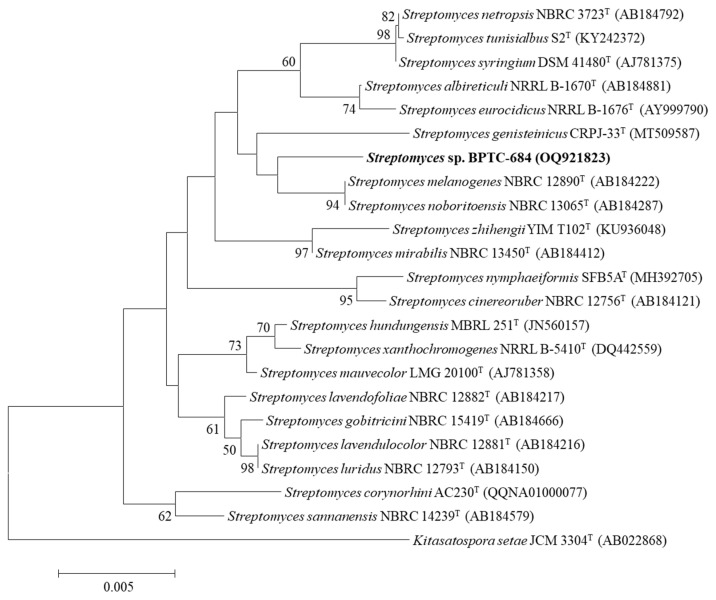
Neighbour-joining tree of strain BPTC-684 and its closely related *Streptomyces* species based on 16S rRNA gene sequencing. Note: Numbers at the nodes represent level of bootstrap support (%) based on a neighbour-joining analysis of 1000 resampled datasets. Only bootstrap values greater than 50% are given. *Kitasatospora setae* JCM 3304^T^ (AB022868) was used as the outgroup. Bar, 0.005 substitutions per site.

The nearly full-length 16S rRNA gene sequence of strain BPTC-684 (GenBank accession number OQ921823; 1526 bp) was compared with the EzBioCloud Database, revealing that strain BPTC-684 had the highest similarity (98.83%) to *S. genisteinicus* CRPJ-33^T^ (MT509587) and *S. melanogenes* NBRC 12890^T^ (AB184222), 98.82% to *S. noboritoensis* NBRC 13065^T^ (AB184287), and 98.18–98.69% to other *Streptomyces* species (Table S2). Concurrently, the phylogenetic tree (Figure 5) also showed that strain BPTC-684 belongs to the genus *Streptomyces* but occupies an independent position relative to its closest relatives. This result implies that strain BPTC-684 is a candidate new species within the genus *Streptomyces*.

To accurately determine the taxonomic position of strain BPTC-684, whole-genome sequencing of the isolate and its closest type strains was performed to simultaneously estimate ANI and dDDH values. The accession numbers for these whole-genome sequences in the NCBI database are mentioned in Table 3. Following this approach, the OrthoANI, ANIb, and dDDH results between strain BPTC-684 and its closest type strains revealed orthology of 78.74–94.88, 77.66–94.38, and 20.20–59.60%, respectively (Table 3). These orthologous ANI and dDDH values were below the cutoffs (<95% for ANI and <70% for dDDH). This suggests that strain BPTC is a candidate new species of the genus *Streptomyces*.

### 3.4. Genome Assembly and Annotation

**Figure 6:**
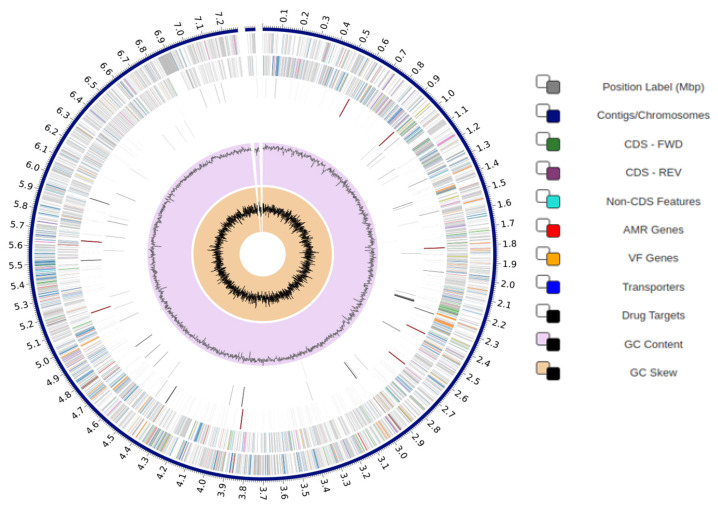
Circular map of *Streptomyces* sp. BPTC-684. Note: CDS—FWD: Coding Sequences Forward; CDS—REV: Coding Sequences Reverse; AMR genes: Antimicrobial Resistance Genes.

The whole genome sequence of strain BPTC-684 was completed using a combination of Illumina MiniSeq and ONT flongle technologies, and was deposited in DDBJ/ENA/GenBank under the accession number CP124863. The chromosome has a length of 7,333,529 bp, with two contigs (the largest, 7,281,262 bp), and a DNA GC content of 71.45%. It has a total of 6881 coding sequences, 213 repeat regions, 23 rRNAs, and 67 tRNAs (Figure 6).

Using antiSMASH revealed 31 biosynthetic gene clusters (BGCs) with a wide range of potential products. Notably, a high-confidence and diverse range of antibiotics were predicted to be produced by the isolate, including kinamycin (belonging to the region 7 gene encoding type II polyketide synthase (T2PKS) and non-ribosomal peptide synthetase-independent siderophore (NI-siderophore)); antimycin (region 27, gene encoding type I polyketide synthase (T1PKS) and non-ribosomal peptide synthetase (NRPS)); fuelimycin A/fuelimicin B/fuelimicin C (region 28, gene encoding T1PKS and prodigiosin); and hangtaimycin/deoxyhangtaimycin (region 30, gene encoding cyclodipeptide synthase (CDPS), trans-AT polyketide synthases (trans AT-PKS)-like, NRPS, PKS-like, and type III polyketide synthase (T3PKS)). Furthermore, siderophores such as the desferrioxamin B/desferrioxamine E gene encoding NI-siderophore (region 16) were also detected (Table 4).

### 3.5. Genes Involved in Plant Growth Promotion

**Figure 7:**
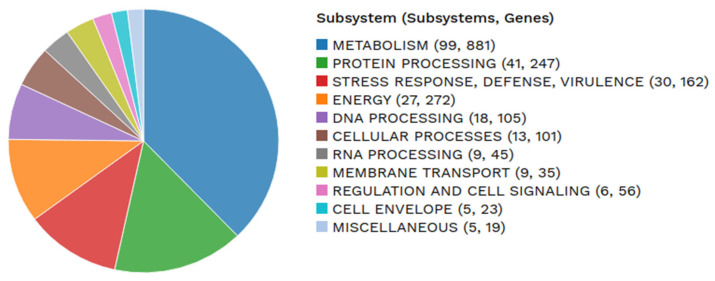
Graphical subsystem category counts of *Streptomyces* sp. BPTC-684 annotated by RASTtk. RASTtk: Rapid Annotations using Subsystems Technology tool kit.

In addition to the potential gene clusters encoding secondary metabolites shown in Table 4, RASTtk analysis of the genome of strain BPTC-684 revealed a diverse range of subsystem features (Figure 7). Notably, 11 genes encoding chitin-degrading enzymes were discovered—including 9 genes encoding chitinase, 1 gene encoding a putative endochitinase (1 gene), and 1 gene encoding a secreted chitinase—along with 33 proteolytic genes including intracellular protease (3 genes), trypsin-like protease (2 genes), putative protease (4 genes), putative metalloprotease (3 genes), metallopeptidase (3 genes), putative peptidase (8 genes), peptidase M48 (2 genes), protease (1 gene), putative secreted protease (1 gene), secreted protease (1 gene), putative dipeptidase (1 gene), peptidase type IV (1 gene), aminopeptidase S (1 gene), putative secreted peptidase (1 gene), and peptidase (1 gene) (Table S3). Chitin and protein are the main components of fungal cells. The presence of these degrading enzymes can inhibit the growth of fungal pathogens affecting corn roots. Simultaneously, genomic strain BPTC-684 also contains the gene encoding indole-3-glycerol phosphate synthase and other functional genes related to tryptophan synthesis and metabolism, implying that IAA biosynthesis in strain BPTC-684 occurs via an independent pathway. Furthermore, the isolate also exhibits a diverse range of genes encoding metabolic products that support plant growth, such as cellulose, amylose, and xylan degradation; urea degradation; phosphorus solubility; and nitrogen metabolism (Table S3).

### 3.6. Impact of Streptomyces sp. BPTC-684 on Treating Fungal Disease in Maize in a Pot Experiment

**Figure 8:**
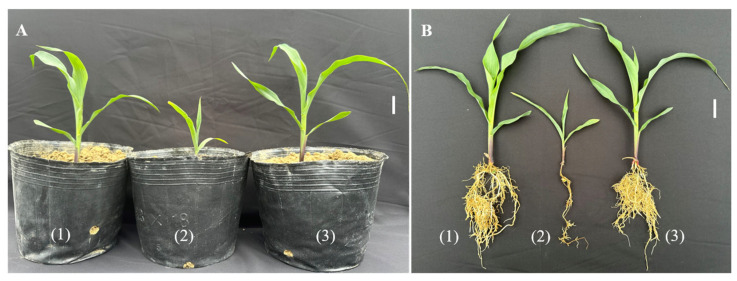
Effect of *Streptomyces* sp. BPTC-684 on maize growth in potted plant experiment on the 15th day. (**A**): Maize grown in pot; (**B**): Morphology characteristic of the maize plant; (1): Control; (2): *F. verticillioides* BNGO-16 inoculation; (3): *Streptomyces* sp. BPTC-684 and *F. verticillioides* BNGO-16 inoculation. Bar, 3 cm.

Pot experiments were conducted to evaluate the ability of *Streptomyces* sp. BPTC-684 as a growth inhibitor to control the fungal disease infecting the plants, as shown in Table 5 and Figure 8. The results revealed no significant differences in plant height, fresh plant weight, or dried root weight between the control and *Streptomyces sp*. BPTC-684 + *F. verticillioides* BNGO-16 inoculation. However, simultaneous infection with *Streptomyces* sp. BPTC-684 and *F. verticillioides* BNGO-16 increased plant height, fresh plant weight, and dry root weight compared to infection with *F. verticillioides* BNGO-16 only, with results of 37.88, 132.50, and 223.81%, respectively (Table 5). This suggests that the presence of *Streptomyces* sp. BPTC-684 supports better maize growth when the maize is infected with the fungal diseases on the 15th day of cultivation in pots.

## 4. Discussion

Controlling maize fungal diseases is essential to prevent yield and quality losses, and contribute to food security for a growing population. These diseases cause serious economic damage, degrade food quality, and produce mycotoxins, necessitating proactive management with biological antagonists [6,51,52] or in combination with sustainable methods such as good agricultural practices [53,54] and fungal-resistant corn [7,55] to maintain healthy, productive agriculture.

*F. verticillioides*, belonging to the *F. fujikuroi* species complex (FFSC), is not only known as the primary infectious agent of wilt and rot in maize [5,6], but also produces dangerous and persistent toxins that seriously contaminate the food chain for humans and livestock [8,9,10,11]. Furthermore, *F. verticillioides* is found in most natural, arid regions and adapts well to adverse conditions [6,13]. Scientists have shown that controlling *F. verticillioides* with antagonistic microorganisms is an increasingly validated solution for agricultural production [6,18,19]. Here, strain BNGO-16, isolated from infected maize roots, exhibits colony morphology in forms of color, surface, and margin that are typical of *F. verticillioides* as described by Nirenberg [56]. The *TEF-1α* shows 99.68% similarity to *F. verticillioides* LC2818 (MW580508) or *F. verticillioides* LC5896 (MW580509) as classified by Chehri et al. 2011 [57]. This value is higher than the cut-off of 99.4% for the same species [58]. The neighbour-joining tree also confirmed this. Therefore, strain BNGO-16 is identified as *F. verticillioides*.

With a more unique genome compared to other Prokaryotes genera, the diversity and abundance of *Streptomyces* species have been classified and their biological potential explored to date [59,60,61]. Our understanding of *Streptomyces* will continue to be strengthened and expanded through new isolated candidates. In this report, strain BPTC-684, isolated from soil using ISP 4 medium supplemented with soil extract [29], demonstrated inhibitory activity of 53.11 ± 2.41% against *F. verticillioides* BNGO-16. Strain BPTC-684 could be considered a candidate new species of the genus *Streptomyces* based on simultaneous data analysis of the differences in cultural characteristics, 16S rRNA gene sequencing, and OrthoANI, ANIb and dDDH values below the thresholds of 95% for ANI [44,45] and 70% for dDDH [43].

The genomic analysis of the novel *Streptomyces* sp. BPTC-684 revealed a G+C content of 71.45% and 31 BGCs; these values fall within the range reported by Belknap et al. [59]. In addition, a diverse range of bioactive compounds, including antibiotics (kinamycin, antimycin, fuelimycins A-C, hangtaimycin, and deoxyhangtaimycin) and siderophores (desferrioxamines B and E), can be produced by strain BPTC-684. Notably, previous reports have shown that kinamycin, a polyketide secondary metabolite (an aminoglycoside antibiotic), known for its potent antimicrobial and cytotoxic properties, often exhibits antifungal activity against agricultural pathogens [62,63]. Reports by Zhu et al. [64] and Park [65] show that antimycin acts as a potent inhibitor of mitochondrial respiration in fungi and other competing microorganisms. It acts as an environmental weapon by disrupting the respiratory chain, inhibiting energy production, and causing rapid cell death. Two bioactive compounds, desferrioxamines B and E, are siderophores (high-affinity iron-chelating compounds) that indirectly inhibit fungi by sequestering essential iron from pathogenic fungi [66,67]. From these arguments it is suggested that kinamycin and antimycin directly participate in the inhibition of *F. verticillioides* BNGO-16 growth, while presence of the remaining bioactive compounds plays an indirect role. As a result, co-involvement of these bioactive compounds may enhance the antifungal efficacy more strongly than that of each compound individually. In addition, *Streptomyces* sp. BPTC-684 produces an enzyme that breaks down chitin, a major component of the fungal cell wall, thereby inhibiting the growth of the pathogenic fungus. During its growth, *Streptomyces* sp. BPTC-684 also exhibited the ability to produce IAA, dissolve phosphorus, and degrade cellulose. Collectively, these traits suggest that *Streptomyces* sp. BPTC-684 enhances maize growth even under *F. verticillioides* infection.

Recent reports indicate that these soil-dwelling bacteria inhibit the fungus through direct antagonistic mechanisms, reducing mycotoxin production and inducing systemic resistance in the crop [20,52,68]. Our findings are consistent with these reports, as BPTC 684 exhibited strong killing of *F. verticillioides* in vitro and promoted better growth of the infected maize in pot experiments.

## 5. Conclusions

In summary, based on differences in cultural characteristics, 16S rRNA gene sequencing, and OrthoANI, ANIb and dDDH values below the thresholds compared to closely related species suggest that BPTC 684 represents a novel *Streptomyces* species. *Streptomyces* sp. BPTC-684 inhibited the growth of *F. verticillioides* BNGO-16 by 53.11%, resulting in increased plant height, fresh weight, and dry root weight by 37.88%, 132.50%, and 223.81%, respectively, in pot experiment infected with the fungus. Therefore, a novel *Streptomyces* sp. BPTC-684 is a potential candidate for the sustainable biological control of fungal diseases and growth promotion in maize.

## Figures and Tables

**Table 1:** Physiological and biochemical characteristics of strain BPTC-684.

Characteristic	Result
Temperature range for growth (°C)	20–40 (optimum 25–30)
pH range for growth	4.0–9.0 (optimum 6.5–7.0)
Growth at NaCl concentration (%, *w*/*v*)	0–7 (optimum 0–4)
IAA production	61.74 ± 2.48 µg/mL
Siderophore production index	5.05 ± 0.11
Phosphate solubilization	+
Extracellular lytic activities:	
Cellulase	+
Chitinase	+
Protease	+
Growth on sole carbon sources (1%, *w*/*v*):	
L-Arabinose	+
D-Xylose	+
D-Fructose	+
Cellulose	+
Rhamnose	w
Raffinose	−
Growth on sole nitrogen sources (0.1% *w*/*v*):	
L-Arginine	−
L-Tyrosine	+
L-Valine	w

−: Negative; +: Positive; w: Weakly positive.

**Table 2:** Cultural characteristics of strain BPTC-684 and closely related species of the genus *Streptomyces* on ISP media.

Characteristic	1	2 ^a^	3	4	5 ^c^	6 ^a^
Yeast extract malt extract agar (ISP 2):						
Aerial spore mass color	Ivory	-	None ^b^	Grey yellow Brown ^b^	-	-
Color of aerial mass	Ivory	Pale olive grey	White ^c^	Grey ^c^	Yellow	Pale smoke grey
Color of reverse mycelium	Pale reddish orange	Old gold	Dark yellow brown ^b^	Dark grey yellow brown ^b^	Yellow brown to greyed yellow	Warm blackish brown
Diffusible pigment	Pale reddish orange	None	None ^b^	None ^b^	None	None
Oatmeal agar (ISP 3):						
Aerial spore mass color	Light grey	-	White ^b^	Yellow white ^b^	-	-
Color of aerial mass	Light grey	Pallid neutral grey	Greyish yellowish pink ^c^	Grey ^c^	Yellow	Court grey
Color of reverse mycelium	Light beige	Viridine green	Dark yellow brown ^b^	Mild yellow brown ^b^	Yellow brown to greyed yellow	Viridine green
Diffusible pigment	None	None	None ^b^	None ^b^	None	None
Inorganic salts starch agar (ISP 4):						
Aerial spore mass color	Light grey	-	Dark yellow brown ^b^	White ^b^	-	-
Color of aerial mass	Light grey	Light mineral grey	Greyish yellowish pink ^c^	Grey ^c^	Yellow	Light mineral grey
Color of reverse mycelium	Pale gold	Pale viridine yellow	Light grey yellow brown ^b^	Dark grey yellow brown ^b^	Yellow brown to greyed yellow	Chrysolite green
Diffusible pigment	Pale gold	None	None ^b^	None ^b^	None	None
Glycerol asparagine agar (ISP 5):						
Aerial spore mass color	None	-	None ^b^	None ^b^	-	-
Color of aerial mass	None	Pale olive grey	None ^c^	Grey ^c^	Yellow	White
Color of reverse mycelium	Yellowish brown	Sulphur yellow	Mild yellow brown ^b^	Dark grey yellow brown ^b^	Yellow brown to greyed yellow	Amber yellow
Diffusible pigment	Yellowish brown	None	None ^b^	None ^b^	None	None
Peptone yeast extract iron agar (ISP 6):						
Aerial spore mass color	None	-	Light grey yellow brown ^b^	None ^b^	-	-
Color of aerial mass	None	White	None ^c^	None ^c^	None	White
Color of reverse mycelium	Grey	Pale orange yellow	Dark grey yellow brown ^b^	Dark grey yellow brown ^b^	Yellow brown to greyed yellow	Capucine yellow
Diffusible pigment	Grey	None	Dark grey yellow brown ^b^	None ^b^	None	None

1: *Streptomyces* sp. BPTC-684 (this study); 2: *S. genisteinicus* CRPJ-33^T^; 3: *S. melanogenes* NBRC 12890^T^; 4: *S. noboritoensis* NBRC 13065^T^; 5: *S. xanthochromogenes* NRRL B-5410^T^; 6: *S. zhihengii* YIM T102^T^. Data was taken from ^a^ Hu et al. [48]; ^b^ Idris et al. [49]; ^c^ Kampfer [50]. -: not known.

**Table 3:** OrthoANI, ANIb, and dDDH values between strain BPTC-684 and its closest related type strains.

Closest Type Strain	NCBI Accession Number	GC Content (%)	OrthoANI (%)	ANIb (%)	dDDH (%)
*S. genisteinicus* CRPJ-33^T^	CP060825	73.39	80.06	79.19	24.00
*S. melanogenes* NBRC 12890^T^	BMTS00000000	71.35	94.78	94.38	59.10
*S. noboritoensis* NBRC 13065^T^	JBHMQV00000000	71.07	94.88	94.47	59.60
*S. xanthochromogenes* NRRL B-5410^T^	BMUZ00000000	71.11	82.55	81.81	26.00
*S. zhihengii* YIM T102^T^	GCA_016919245	72.87	81.43	79.27	24.10
*S. albireticuli* NRRL B-1670^T^	NSJV00000000	72.42	78.92	77.82	23.30
*S. mauvecolor* LMG 20100^T^	BAAASQ010000000	70.86	82.94	82.18	26.40
*S. eurocidicus* NRRL B-1676^T^	LGUI00000000	72.62	78.74	77.66	23.00
*S. netropsis* NBRC 3723^T^	BMRW00000000	71.56	78.76	77.75	20.20

ANI: Average Nucleotide Identity; ANIb: Average Nucleotide Identity based on BLAST v2.2.29^+^; dDDH: digital DNA–DNA hybridization. ANI ≥ 95% and dDDH ≥ 70% are the cutoff thresholds for the same species, respectively.

**Table 4:** Predicting biosynthetic gene clusters (BGCs) for genome assembly of strain BPTC-684 using the antiSMASH v.8.0.4 tool.

Region	Type	From	To	Similarity Confidence	Most Similar Known Cluster
1	Terpene	80,786	107,363	High	Hopene
2	T1PKS, hglE-KS	134,846	186,081	-	-
3	Redox-cofactor, T3PKS	252,577	306,071	-	-
4	PKS-like	453,156	498,998	-	-
5	RiPP-like	591,124	613,671	Low	14-hydroxyisochainin
6	Hydrogen-cyanide	653,362	666,355	Low	Aborycin
7	T2PKS, NI-siderophore	820,681	899,701	High	Kinamycin
8	CDPS	1,268,632	1,289,354	Low	BD-12
9	Terpene-precursor	1,593,095	1,614,093	-	-
10	Lanthipeptide-class-i	1,758,239	1,782,673	-	-
11	T3PKS	2,654,167	2,695,264	-	-
12	Terpene	2,793,620	2,815,851	Low	TVA-YJ-2
13	NRPS-like	3,184,572	3,227,172	Low	Arginomycin
14	Melanin	3,530,627	3,541,001	Medium	4-hydroxy-3-nitrosobenzamide
15	Phenazine	3,564,510	3,584,998	Low	Diastaphenazine/Izumiphenazine C
16	NI-siderophore	4,078,823	4,108,601	High	Desferrioxamin B/Desferrioxamine E
17	Butyrolactone	4,356,331	4,367,224	Low	Coelimycin P1
18	Lanthipeptide-class-iii	4,516,914	4,539,559	Medium	SapB
19	Other	4,870,174	4,910,590	Low	Stlassin
20	Ectoine	5,037,378	5,047,782	High	Ectoine
21	RiPP-like	5,826,550	5,836,777	-	-
22	T2PKS	5,923,776	5,996,372	Medium	Alnumycin A/alnumycin B/Alnumycin C/Alnumycin P/Prealnumycin/Thalnumycin A/Thalnumycin B/K1115A/1,6-dihydro-8-propylanthraquinone
23	Melanin	6,098,043	6,108,471	Low	Melanin
24	Terpene	6,121,351	6,142,412	High	Pristinol
25	Lanthipeptide-class-iv	6,169,605	6,192,400	-	-
26	T3PKS	6,203,966	6,245,003	High	Flaviolin/1,3,6,8-tetrahydroxynaphthalene
27	T1PKS, NRPS	6,268,196	6,350,117	High	Antimycin
28	T1PKS, prodigiosin	6,554,889	6,635,104	High	Fuelimycin A/Fuelimicin B/Fuelimicin C
29	Lanthipeptide-class-iv, terpene	6,775,364	6,801,729	-	-
30	CDPS, trans-AT-PKS-like, NRPS, PKS-like, T3PKS	6,816,409	6,932,063	High	Hangtaimycin/Deoxyhangtaimycin
31	Terpene	7,127,915	7,148,721	-	-

NRPS: Non-ribosomal peptide synthetase; NRPS-like: NRPS-like fragment; T1PKS: Type I polyketide synthase; T2PKS: Type II polyketide synthase; T3PKS: type III polyketide synthase; RiPP-like: Ribosomally synthesized and Post-translationally modified peptides; CDPS: Cyclodipeptide synthase; trans-AT-PKS-like: trans-acyltransferase polyketide synthases; NI-siderophore: Non-Ribosomal Peptide Synthetase-Independent Siderophore; -: not detected.

**Table 5:** Comparison of promoting plant growth in the potted plant experiment.

Treatment	Plant Height (cm)	Fresh Plant Weight (g)	Dry Root Weight (g)
Control	25.08 ± 1.81 ^a^	1.01 ± 0.13 ^a^	0.74 ± 0.15 ^a^
BNGO-16 inoculation	17.74 ± 1.74 ^b^	0.40 ± 0.09 ^b^	0.21 ± 0.07 ^b^
BPTC-684 and BNGO-16 inoculation	24.46 ± 1.65 ^a^	0.93 ± 0.16 ^a^	0.68 ± 0.11 ^a^

Different letters in the same column indicate statistically significant differences at the *p* ≤ 0.05 by using JASP 0.95.4 software. Data are presented as mean values ± standard deviation (SD).

## Data Availability

The original contributions presented in this study are included in the article/Supplementary Material. Further inquiries can be directed to the corresponding author.

## Supplementary Materials

The following supporting information can be downloaded at: https://www.mdpi.com/article/10.3390/microorganisms14040818/s1. Table S1: Characteristics of strain BPTC-684 grown on various media after 21 days of incubation at 28 °C; Table S2: Comparison of 16S rRNA gene sequence of strain BPTC-684 with the closest type strains; Table S3: Genes involved in plant growth-promoting traits were detected in the genome of strain BPTC-684.
