# Supplementary material for: Identification of Novel Streptomyces sp. BPTC-684 as a Biocontrol Agent Against Challenging Maize Root Rot Caused by Fusarium verticillioides
Source: Microorganisms. 2026 Apr 2;14(4):818. doi: 10.3390/microorganisms14040818 (PMC13119414; doi:10.3390/microorganisms14040818)
Supplement: Supplementary file 1 [file microorganisms-14-00818-s001.zip › microorganisms-4211097-supplementary.pdf]

Supplementary Tables

Table S1. Characteristics of strain BPTC-684 grown on various media after 21 days of incubation at 28°C

Medium	Growth	Sporulation	Color of: Aerial mass	Color of: Reverse mycelium	Diffusible pigment
Czapek–Doxagar	Poor	Absent	Orange yellow	None	None
Potato dextrose agar	Poor	Good	Light grey	None	None
Nutrient agar	Abundant	Absent	Ivory	None	None
Trypticase soy agar	Good	Absent	Orange yellow	Orange yellow	Orange yellow
Yeast extract malt extract agar (ISP 2)	Abundant	Moderate	Ivory	Pale reddish orange	Pale reddish orange
Oatmeal agar (ISP 3)	Abundant	Good	Light grey	Light beige	None
Inorganic salts starch agar (ISP 4)	Abundant	Good	Light grey	Pale gold	Pale gold
Glycerol asparagine agar (ISP 5)	Abundant	None	None	Yellowish brown	Yellowish brown
Peptone yeast extract iron agar (ISP 6)	Abundant	None	None	Grey	Grey

Table S2. Comparison of 16S rRNA gene sequence of strain BPTC-684 with the closest type strains

**The most closely related species**	**Pairwise Similarity (%)**	**Mismatch/Total nucleotide**
Streptomyces genisteinicus CRPJ-33^T (MT509587)	98.83	17/1448
Streptomyces melanogenes NBRC 12890^T (AB184222)	98.83	17/1447
Streptomyces noboritoensis NBRC 13065^T (AB184287)	98.82	17/1446
Streptomyces xanthochromogenes NRRL B-5410^T (DQ442559)	98.69	19/1448
Streptomyces zhihengii YIM T102^T (KU936048)	98.69	19/1448
Streptomyces albireticuli NRRL B-1670^T (AB184881)	98.69	19/1445
Streptomyces mauvecolor LMG 20100^T (AJ781358)	98.62	20/1447
Streptomyces eurocidicus NRRL B-1676^T (AY999790)	98.62	20/1446
Streptomyces nymphaeiformis SFB5A^T (MH392705)	98.48	22/1448
Streptomyces netropsis NBRC 3723^T (AB184792)	98.48	22/1445
Streptomyces tunisialbus S2^T (KY242372)	98.43	22/1398
Streptomyces hundungensis MBRL 251^T (JN560157)	98.41	23/1448
Streptomyces lavendofoliae NBRC 12882^T (AB184217)	98.41	23/1446
Streptomyces syringium DSM 41480^T (AJ781375)	98.41	23/1445
Streptomyces cinereoruber NBRC 12756^T (AB184121)	98.41	23/1443
Streptomyces corynorhini AC230^T (QQNA01000077)	98.27	25/1448
Streptomyces gobitricini NBRC 15419^T (AB184666)	98.20	26/1447
Streptomyces mirabilis NBRC 13450^T (AB184412)	98.20	26/1447
Streptomyces luridus NBRC 12793^T (AB184150)	98.18	26/1430
Streptomyces lavendulocolor NBRC 12881^T (AB184216)	98.18	26/1429
Streptomyces sannanensis NBRC 14239^T (AB184579)	98.18	26/1429

Table S3. Genes involved in plant growth-promoting found in the genome of strain BPTC-684

Subsystem category	Coding Sequence	Start	Stop	Feature_id
IAA biosynthesis	Tryptophan 2,3-dioxygenase (EC 1.13.11.11)	3173865	3174743	fig|1883.2928.peg.2939
	Kynureninase (EC 3.7.1.3)	3174736	3175926	fig|1883.2928.peg.2940
	Indole-3-glycerol phosphate synthase (EC 4.1.1.48)	4832247	4833056	fig|1883.2928.peg.4447
	Tryptophan synthase beta chain (EC 4.2.1.20)	4833356	4834603	fig|1883.2928.peg.4449
	Tryptophan synthase alpha chain (EC 4.2.1.20)	4834600	4835415	fig|1883.2928.peg.4450
	Tryptophanase (EC 4.1.99.1)	7061473	7062849	fig|1883.2928.peg.6570
Siderophore biosynthesis	Siderophore synthetase component, ligase	882512	880530	fig|1883.2928.peg.785
	Siderophore synthetase small component, acetyltransferase	883703	882573	fig|1883.2928.peg.786
	Siderophore synthetase superfamily, group A Siderophore synthetase large component, acetyltransferase	885701	883743	fig|1883.2928.peg.787
	Siderophore biosynthesis diaminobutyrate-2-oxoglutarate aminotransferase (EC 2.6.1.76)	887139	885730	fig|1883.2928.peg.788
	Siderophore biosynthesis protein, monooxygenase	2789309	2788014	fig|1883.2928.peg.2570
	Desferrioxamine E biosynthesis protein Des D Siderophore synthetase superfamily, group C Siderophore synthetase component, ligase	4094601	4092823	fig|1883.2928.peg.3744
	Desferrioxamine E biosynthesis protein Des C Siderophore synthetase small component, acetyltransferase	4095140	4094598	fig|1883.2928.peg.3745
	Desferrioxamine E biosynthesis protein Des B Siderophore biosynthesis protein, monooxygenase	4096411	4095137	fig|1883.2928.peg.3746
	Desferrioxamine E biosynthesis protein Des A Siderophore biosynthesis L-2,4-diaminobutyrate decarboxylase	4097837	4096392	fig|1883.2928.peg.3747
	Siderophore biosynthesis L-2,4-diaminobutyrate decarboxylase	4896617	4897981	fig|1883.2928.peg.4507
	Siderophore biosynthesis protein, monooxygenase	4897978	4899390	fig|1883.2928.peg.4508
	ABC-type Fe^{3+}-siderophore transport system, permease 2 component	5161903	5163414	fig|1883.2928.peg.4773
	ABC-type Fe^{3+}-siderophore transport system, ATPase component	5163468	5163995	fig|1883.2928.peg.4774

	Isochorismatase (EC 3.3.2.1) of siderophore biosynthesis	5968381	5967737	fig|1883.2928.peg.5529
	Thioesterase in siderophore biosynthesis gene cluster	6842840	6842097	fig|1883.2928.peg.6394
	Siderophore synthetase component, ligase	882512	880530	fig|1883.2928.peg.785
	Siderophore synthetase small component, acetyltransferase	883703	882573	fig|1883.2928.peg.786
	Siderophore synthetase superfamily, group A Siderophore synthetase large component, acetyltransferase	885701	883743	fig|1883.2928.peg.787
	Siderophore biosynthesis diaminobutyrate--2-oxoglutarate aminotransferase (EC 2.6.1.76)	887139	885730	fig|1883.2928.peg.788
	Siderophore biosynthesis protein, monooxygenase	2789309	2788014	fig|1883.2928.peg.2570
Degradation of chitin	Chitinase (EC 3.2.1.14)	257958	256957	fig|1883.2928.peg.234
		1027023	1025161	fig|1883.2928.peg.906
		1425912	1427771	fig|1883.2928.peg.1274
		1871202	1872893	fig|1883.2928.peg.1722
		4056134	4057846	fig|1883.2928.peg.3711
		5572503	5571268	fig|1883.2928.peg.5163
		6036706	6034400	fig|1883.2928.peg.5590
		6500368	6498629	fig|1883.2928.peg.6036
		6765392	6767635	fig|1883.2928.peg.6309
	Putative endochitinase	5833210	5832158	fig|1883.2928.peg.5394
	Secreted chitinase (secreted protein)	6367099	6365585	fig|1883.2928.peg.5899
Degradation of cellulose	Beta-1,4-glucanase (cellulase) (EC 3.2.1.4)	256947	255685	fig|1883.2928.peg.233
Degradation of amylose	Alpha-amylase (EC 3.2.1.1)	1315331	1317040	fig|1883.2928.peg.1173
	Glucoamylase (EC 3.2.1.3)	5083996	5082215	fig|1883.2928.peg.4673
		5174169	5173111	fig|1883.2928.peg.4786
		5175033	5174353	fig|1883.2928.peg.4787
	Putative alpha-amylase	6013772	6015199	fig|1883.2928.peg.5575
Degradation of xylan	Endo-1,4-beta-xylanase (EC 3.2.1.8)	6756883	6755513	fig|1883.2928.peg.6302
Degradation of protein	Intracellular protease	109912	109277	fig|1883.2928.peg.102
		3872629	3872063	fig|1883.2928.peg.3552
		5835568	5834870	fig|1883.2928.peg.5397
	Trypsin-like protease	803009	802179	fig|1883.2928.peg.717
		4883944	4884726	fig|1883.2928.peg.4498
	Putative protease	893383	891938	fig|1883.2928.peg.792
		2554288	2552738	fig|1883.2928.peg.2380
		3650444	3647157	fig|1883.2928.peg.3370
		5096468	5094948	fig|1883.2928.peg.4685
	Protease	2605788	2606921	fig|1883.2928.peg.2425
	Putative metalloprotease	1263405	1261999	fig|1883.2928.peg.1137
		1539264	1536400	fig|1883.2928.peg.1381
		4355824	4354763	fig|1883.2928.peg.3955
	Metallopeptidase	3336530	3337921	fig|1883.2928.peg.3098

		6462347	6464197	fig|1883.2928.peg.5997
		126775	128274	fig|1883.2928.peg.116
	Putative secreted protease	3989611	3992019	fig|1883.2928.peg.3642
	Secreted protease	4689933	4688824	fig|1883.2928.peg.4310
	Putative dipeptidase	579887	578757	fig|1883.2928.peg.532
	Putative peptidase	779152	778382	fig|1883.2928.peg.694
		1750395	1752524	fig|1883.2928.peg.1622
		2435015	2434365	fig|1883.2928.peg.2264
		2671257	2670178	fig|1883.2928.peg.2473
		3243163	3241358	fig|1883.2928.peg.3003
		4436641	4437792	fig|1883.2928.peg.4049
		5680856	5678757	fig|1883.2928.peg.5252
		779152	778382	fig|1883.2928.peg.694
	Peptidase M48	668857	669819	fig|1883.2928.peg.603
		2244839	2243976	fig|1883.2928.peg.2094
	Peptidase type IV	1857354	1856656	fig|1883.2928.peg.1702
	Aminopeptidase S (EC 3.4.11.26)	2268162	2269481	fig|1883.2928.peg.2115
	Putative secreted peptidase	4434991	4435887	fig|1883.2928.peg.4047
	Peptidase	80062	79118	fig|1883.2928.peg.75
Ure utilization	Urease alpha subunit (EC 3.5.1.5)	1203805	1202072	fig|1883.2928.peg.1083
		5775452	5777173	fig|1883.2928.peg.5346
	Urease gamma subunit (EC 3.5.1.5)	1204506	1203802	fig|1883.2928.peg.1084
		5774829	5775131	fig|1883.2928.peg.5344
	Urease beta subunit (EC 3.5.1.5)	5775148	5775459	fig|1883.2928.peg.5345
	Urease accessory protein UreF	5777209	5777883	fig|1883.2928.peg.5347
	Urease accessory protein UreG	5778178	5778855	fig|1883.2928.peg.5348
	Urease accessory protein UreD	5778843	5779625	fig|1883.2928.peg.5349
Solubilization of phosphorus	Alkaline phosphatase (EC 3.1.3.1)	3245245	3246774	fig|1883.2928.peg.3006
	Inorganic pyrophosphatase (EC 3.6.1.1)	2483395	2483889	fig|1883.2928.peg.2309
	Phosphodiesterase/alkaline phosphatase D	4493617	4493090	fig|1883.2928.peg.4131
		4494331	4493642	fig|1883.2928.peg.4132
		4494798	4494385	fig|1883.2928.peg.4133
		5741427	5739844	fig|1883.2928.peg.5306
	Exopolyphosphatase (EC 3.6.1.11)	3746043	3746957	fig|1883.2928.peg.3445
	Acid phosphatase (EC 3.1.3.2)	4613285	4612395	fig|1883.2928.peg.4246
		6526527	6527450	fig|1883.2928.peg.6066
	Putative secreted alkaline phosphatase	4805843	4804194	fig|1883.2928.peg.4419
	Putative phosphatase	6511526	6512176	fig|1883.2928.peg.6049
Nitrogen metabolism	Nitrite reductase [NAD(P)H] large subunit (EC 1.7.1.4)	4407051	4406389	fig|1883.2928.peg.4009
		4409248	4407089	fig|1883.2928.peg.4010
	Assimilatory nitrate reductase large subunit (EC 1.7.99.4)	691461	696008	fig|1883.2928.peg.622
		3607067	3609361	fig|1883.2928.peg.3338
	Glutamine synthetase type I (EC 6.3.1.2)	4607780	4609141	fig|1883.2928.peg.4241
		4653556	4652147	fig|1883.2928.peg.4279
	Glutamine synthetase type II (EC 6.3.1.2)	4648229	4647192	fig|1883.2928.peg.4273
	Glutamine synthetase (EC 6.3.1.2), putative	5532227	5530899	fig|1883.2928.peg.5127
